# I love you ... and heroin: care and collusion among drug-using couples

**DOI:** 10.1186/1747-597X-1-7

**Published:** 2006-03-28

**Authors:** Janie Simmons, Merrill Singer

**Affiliations:** 1 National Development and Research Institutes/ Medical and Health Research Association of NYC, NY, NY, USA; 2 Hispanic Health Council, Inc. Hartford, CT, USA

## Abstract

**Background:**

Romantic partnerships between drug-using couples, when they are recognized at all, tend to be viewed as dysfunctional, unstable, utilitarian, and often violent. This study presents a more nuanced portrayal by describing the interpersonal dynamics of 10 heroin and cocaine-using couples from Hartford, Connecticut.

**Results:**

These couples cared for each other similarly to the ways that non-drug-using couples care for their intimate partners. However, most also cared by helping each other avoid the symptoms of drug withdrawal. They did this by colluding with each other to procure and use drugs. Care and collusion in procuring and using drugs involved meanings and social practices that were constituted and reproduced by both partners in an interpersonal dynamic that was often overtly gendered. These gendered dynamics could be fluid and changed over time in response to altered circumstances and/or individual agency. They also were shaped by and interacted with long-standing historical, economic and socio-cultural forces including the persistent economic inequality, racism and other forms of structural violence endemic in the inner-city Hartford neighborhoods where these couples resided. As a result, these relationships offered both risk and protection from HIV, HCV and other health threats (e.g. arrest and violence).

**Conclusion:**

A more complex and nuanced understanding of drug-using couples can be tapped for its potential in shaping prevention and intervention efforts. For example, drug treatment providers need to establish policies which recognize the existence and importance of interpersonal dynamics between drug users, and work with them to coordinate detoxification and treatment for both partners, whenever possible, as well as provide additional couples-oriented services in an integrated and comprehensive drug treatment system.

## Background

### Introduction

Nina Glick-Schiller [1] aptly captured the dehumanization and distortion of relatively stable intimate partnerships among drug-users when she wrote, "While other people have lovers and spouses, drug users have only 'sex partners."' This ethnographic study contributes to a broader, more nuanced portrayal of drug users by describing the interpersonal dynamics of 10 heroin and cocaine-using couples from Hartford, Connecticut. These couples cared for each other similarly to the ways that non-drug-using couples care for their intimate partners. However, most also cared by helping each other avoid the symptoms of withdrawal. They did this by colluding with each other to procure and use drugs. Care and collusion in procuring and using drugs involved meanings and social practices that were constituted and reproduced by both partners in an interpersonal dynamic that was often overtly gendered. These gendered dynamics could be fluid and changed over time in response to altered circumstances and/or individual agency. They also were shaped by and interacted with long-standing historical, economic and socio-cultural forces including the persistent economic inequality, racism and other forms of "structural violence" [2,3] endemic in the inner-city Hartford neighborhoods where these couples resided. As a result, these relationships offered both risk and protection from HIV, HCV and other health threats (e.g. arrest and violence). An understanding of the complex interpersonal dynamics between drug-using couples has not, as yet, been tapped for its potential to shape prevention and intervention efforts that would reduce drug use, HIV and other health risks faced by this population. Based on the findings reported here, we suggest that drug treatment providers recognize the existence and importance of interpersonal dynamics between drug users, and work with them to coordinate detoxification and treatment for both partners, whenever possible, as well as provide additional couples-oriented services in an integrated and comprehensive drug treatment system.

### HIV prevention research and injecting drug-using couples

AIDS prevention research among drug users was initially dominated by a set of psychological models of motivation and behavioral change which placed a disproportionate emphasis on individual cognitive and motivational variables, rather than social and structural influences on individual behavior [4]. However, this narrow focus on the individual as an independent force in behavior was restrictive and failed to fully consider micro-social (i.e., network) and macro-social (i.e., larger social context) factors [5-7] that shape rates, routes, and patterns of HIV infection [8]. Consequently, a number of researchers incorporated social network concepts and analyses in the study of drug use and disease transmission among drug users [5,9,10]. Social network research has consistently demonstrated that underlying network characteristics shape the pattern and rate of diffusion of HIV in local contexts [11]. While network research has advanced our understandings of HIV transmission, one limitation of this research among drug users is the tendency to submerge all relationships into the study of network structure, characteristics and behavioral patterns. The meanings of significant dyadic relationships within networks, such as intimate partnerships, and the behavioral implications of these meanings, however, tend to be overlooked in network studies.

While research on intimate partnerships among injection drug users (IDUs) is still sparse, some studies of differences between men and women IDUs are available. For example, prevention research pointed out the heightened risk that women IDUs face because they are more likely than male IDUs to acquire HIV sexually [12,13] and to have sexual partners who are injecting drug users [14-22]. Prevention research among drug users also has consistently demonstrated how socially embedded meanings tied to intimacy, such as trust, may lower the perception of risk attached to needle sharing and unprotected sex [19,23,24].

MacRae and Aalto [25] interviewed women IDUs in Scotland and found that women's patterns of injecting and needle sharing were strongly influenced by the nature and type of their sexual relationships because women tended to place a high level of importance on their intimate partnerships and invested significantly in them. Among the drug-using women in their study, almost all shared needles with their partners and most were injected by their partners. Citing Barnard [19], they also emphasize that "the interplay of social factors such as the distribution of power and control, particularly regarding the division of money and drugs between injecting couples, may influence the way HIV risks are managed."

Rhodes and Quirk [26] appear to be the first HIV prevention researchers to identify injecting-drug-using couples as an important unit of analysis. Their qualitative study of couples in London focused on the influence of primary sexual relationships in the lives of drug users. They illustrated how drug users' sexual relationships serve as key sites for risk management in individuals' drug use and everyday lifestyles. They also found that relationships among drug users tended to have an "equalizing effect" on patterns of drug use as a function of sharing the money to buy drugs and sharing the drugs themselves. As a result, drug consumption in these relationships increased rather than decreased. Attempts to reduce or stop drug use were avoided or hard to sustain because they threatened the stability of the relationship.

### Co-dependency, enabling and intimate relationships among drug users

Cavacuiti [27] recently surveyed the non-HIV research literature on couples and substance abuse and noted a primary focus on "codependency." Almost exclusively, these studies focus on alcoholics and their non-drinking spouses (usually wives). With respect to couples where both partners use drugs, he [27] asserts, "It cannot be said that the dearth of literature on this subject exists because such relationships do not exist." Codependency theory has been widely criticized and its applicability to research on illicit-drug-using couples, especially when both partners use substances, is questionable [28-31]. Nevertheless, codependency theory, in its most popular form, is often supported by advocates of self-help groups such as Alcoholics Anonymous (AA) and Narcotics Anonymous (NA), as well as detoxification and treatment program staff who work in programs based on the philosophies and methods of these groups [32]. Generally, co-dependency theory assumes that "family members of alcoholics and addicts are part of a dysfunctional family system, and that they, unwittingly, contribute to the perpetration of the alcoholic's or addict's destructive behavior" [32]. (See also Steinglass [33]).

More psychoanalytically derived theories of co-dependency deal with the need for obtaining and preserving affection, despite the cost of being dependent and exploited [32]. Critics of codependency theory have observed that "many of the behaviors described as codependent ... are behaviors traditionally ascribed to women who, more so than men, think of close relationships and caring for others as healthy, adaptive, and integral to the human experience"[32]. Rotunda and Doman [32] also note that researchers attempting to avoid the pitfalls of co-dependency theory have opted for the term "enabling" in order to place the focus on the behaviors of the alcoholic spouse or family member who either enables or disables the substance user to make a positive change (reduce drinking, stop drinking, etc.). Enabling theory, like co-dependency, however, focuses on non-substance-using spouses and other family members of a substance user, rather than the dyad itself.

The earliest studies on the interpersonal dynamics of couples where both partners use illicit substances tended to mirror the codependency and enabling literature in the sense that these relationships were seen as dysfunctional but utilitarian in the acquisition of drugs. For example, Rosenbaum, in her 1981 seminal study, Women on Heroin, writes:

Heroin use becomes the focal point of the relationship and erodes other aspects of affection and mutuality; the heroin life disrupts traditional sex role delineation to the dissatisfaction of the couple; and unscrupulousness and money problems cause nearly constant bickering [34].

Inciardi (1981) utilized the same construct in Women and Crack Cocaine:

With increased time as a heroin or cocaine street addict ... comes an increased probability that even still existing personal relationships will be terminated – marriages end in large part due to fighting about drug use, children are taken away by courts or conventional relatives. In short, a woman's participation in the ultimate drug involvement of the heroin/cocaine lifestyle very often leads to a situation which is the ultimate in social isolation [35].

Although Judith Porter [36] found less of a tendency to terminate intimate relationships she identified a similar pattern of dependence and dysfunctionality. Porter noted that relationships among heroin-addicted partners tend to be unstable and abusive, as well as pragmatic in nature. Women depended on men for drugs or money to buy drugs.

### Focusing on couples as a unit of analysis

HIV prevention research among drug-using intimates has focused on the dynamics of transmission between drug-using men and women, the distribution of risk in intimate relationships and the ways in which risk is managed in these relationships. However, researchers have not adequately examined ways in which both men and women make sense of and experience these relationships. Similarly, with the exception of Rhodes's and Quirk's [26] study of drug-using couples in London, couples have not been utilized as a unit of analysis.

Some studies focused on drug-using women have begun to shed light on relationship-dynamics [37-39]. For example, one noteworthy development in treatment research is the finding that women who report higher relationship quality were less likely to complete treatment and more likely to use drugs post-treatment than women who reported lower relationship quality [39]. In another development involving research on couples, it was found that relationship factors can have a strong positive impact on both sexual risk behavior and quality of life as measured by enrollment in HAART, health status, and psychological well-being among drug-using couples in treatment [40].

Further study is needed, particularly of out-of-treatment, drug-using couples, in order to gain a more comprehensive and accurate understanding of the varied and complex interpersonal dynamics which characterize these relationships, and how these dynamics are shaped by larger forces, including the inability of treatment programs for the poor to adequately deal with polydrug use in general, and couples, in particular. This study was intended to address these issues.

## Methods

### Study site

This study was conducted in 2000 at the Hispanic Health Council, Inc. (HHC), a community-based research and advocacy organization with a long history of involvement in research and intervention projects with illicit drug users in Hartford, Connecticut. Although Hartford is the capital of the wealthiest state per capita in the country, it is the poorest city in the state and one of the nation's poorest per capita for moderate size cities. It has a population of approximately 125,000. According to the 2000 Census, whites comprise 18% of the population, African Americans (including many Caribbeans of African descent) 38% and Hispanics 40%. Puerto Ricans comprise 89% of the Hispanic population, making it the most Puerto Rican city per capita in the United States [41]. Hartford's segregated neighborhoods are similar to those described by Wallace in NYC as "environments of risk" where urban poverty and political ill will combine to create a "synergy of plagues" [42,43]. Building on findings among drug users in Hartford, a parallel argument was developed by Singer and Clair [44]. They describe the relationship between social environment and disease interaction as a critical factor magnifying the disease burden of the poor and other marginalized populations.

During the study period, drug injection accounted for one half of all new HIV infections and was the most frequent source of new HIV infections nationwide (1.5 infections per 100 injecting drug users per year). Most of these new infections were occurring in cities in the Northeast [45] where AIDS had been the leading cause of death between men and women aged 24–45 years of age. In Connecticut in general, and Hartford specifically, drug use was related to an even larger percentage of AIDS cases than in the nation as a whole. Intravenous drug users, their heterosexual "sex" partners, and their children constituted 61% of the total AIDS cases in the State, as compared to 35% nationally, and 44% in the Northeast. Hartford had annual AIDS rates of 37.1 per 100,000, placing it among the 50 U.S. cities with the highest annual AIDS rates [46]. A new study has estimated that nearly 13% of Hartford's IDUs were HIV positive during this period. This rate of infection ranked Hartford 14^th ^among the largest metropolitan areas in the U.S. [47]. An authoritative estimate of the numbers of IDU's in the Hartford area places this hidden population at 9,600 [48].

### Study sample

A total of 10 drug-using couples were recruited through street outreach. Two criteria for recruitment were followed for eight of the 10 couples: At least one member of the couple had to be an injection drug user and both members had to use heroin, cocaine, or both on a daily basis. In two cases, couples were admitted after they were enrolled in methadone maintenance programs. In both cases, these couples used heroin on occasion but were not daily users. In addition, each member of the couple had to define themselves as a couple and have been sexually involved for at least 6 months. Comparative data which characterizes these 10 couples appear in Table 1.

**Table 1:** Couples Demographics.

	Race/Ethnicity	Gender	Length of Relationship	Residence	Drug Use	HIV/HEP Status
CO1: DIANA	B	F	6	APT, HOMELESS	H, C, CR, A	HIV+, HEP C
CO1: GLENN	B	M	6	APT, HOMELESS	H, C, CR, A	HIV+

CO2: SANDRA	PR	F	5	APT	H	HIV-
CO2: JULIO	PR	M	5	APT	H	HIV- HEP C

CO3: DAISY	PR	F	11	ALT W/ FAMILY, HOMELESS	H, A	HIV+
CO3: JUAN	PR	M	11	ALT W/ FAMILY, HOMELESS	H	HIV+

CO4: PATRICIA	PR	F	4	APT	H,	HIV+
CO4: ANDRES	PR	M	4	APT	H,	HIV+

CO5: LILIA	PR	F	7	APT, HOMELESS, APT	H	HIV-
C05: REINALDO	PR	M	7	APT, HOMELESS, APT	H	HIV-

CO6: RAQUEL	PR	F	22	APT W/ 2 CHILDREN	H	HIV-
CO6: VICENTE	PR	M	22	APT W/ 2 CHILDREN	H (SNIFFS)	HIV-

CO7: CANDY	W	F	4	HOMELESS, APT	H	HIV-
CO7: LEONARDO	PR	M	4	HOMELESS, APT	C,	HIV-

CO8: OLIVIA	PR	F	2	HOMELESS	H	HIV-
CO8: SANTO	PR	M	2	HOMELESS	H	HIV-

CO9: ALTHEA	B	M	3	APT	H, A,	HIV+ HEP C
CO9: GEORGE	B	F	3	APT	H	HIV+

C10: DOUGLAS	B	M	7	APT, HOMELESS, APT	H, C, CR, A	HIV+
C10: CHRISTOPHER	B	M	7	APT, HOMELESS, APT	H, C, CR, A	HIV+

All names are pseudonyms. H = heroin; C = cocaine; CR = crack; A = alcohol. Apt, homeless, Apt means during the course of the study, couples moved from an apartment to being homeless, to an apartment again.

As depicted in the table, 12 partners in the study injected heroin only or injected both heroin and cocaine (7 partners). The sole non-injector was a woman who sniffed heroin. The duration of their partnerships ranged from 2 to 22 years (mean = 7.1) at the beginning of the study period. Most of the couples had been together from 3 to 7 years. Three of the couples were African-American, six were Puerto Rican, and one couple was Puerto Rican and white. Their ages ranged from 30 to 51 (most were in their 30's or 40's). Nine of the ten couples were partnered in heterosexual unions. None were legally married. One couple paired two men. Half of the individuals reported being HIV and HCV positive. All couples shared the same serostatus for HIV (1/2 were HIV+, half were HIV-). Three individuals, in three separate couples, reported HCV positivity, but most did not know their serostatus for HCV.

### Procedures

The Institutional Review Boards at the Hispanic Health Council, Inc. and Yale University approved the project and a certificate of confidentiality was granted by the federal government. Semi-structured, two-on-one, couple interviews were conducted with each dyad after reading and signing consent forms which clearly described the study as a "couples study." These first interviews explored partnership history, interaction patterns, drug-use behaviors, and AIDS risk behavior. In addition, one-on-one, in-depth, open-ended interviews were conducted with each partner about the nature of the relationship; drug treatment attitudes and enrollment efforts; levels of conflict and support within the relationship; survival and "hustling" strategies employed by partners; history of and current drug use; and HIV risk behaviors with their primary partners and others.

Whenever possible, ethnographic observations were conducted to reveal the day-to-day realities and contexts of risk of drug-using couples. These included observations of drug-use, "hustling" strategies, public interactions with partners and others, and use or non-use of risk-reduction strategies (including needle-exchange). Fieldnotes were written after each observation and included a narrative profile of each individual or dyad; the context(s) of the interaction; others who may have been present; a detailed accounting of drug use and specific risk behaviors observed; the content of the conversations; the primary concerns of the couple; and the relationship of these observed events, activities and behaviors with other observed or recorded events and behaviors. Study participants received $15 in compensation for interviews and observations.

A grounded theory approach [49] was used to inductively code and analyze the nearly 40 1–1 1/2 hour transcripts and fieldnote data with the aid of Atlas qualitative coding software [50]. Findings were analyzed for patterns and associations between characteristics of intimate dyads and their impact on drug use, drug treatment, HIV risk, and other health risks. While the study itself lasted a year, the data accumulated about these couples were gathered over a longer period. Some individuals participated in other Hispanic Health Council studies involving the first author and otherwise sought her out for referrals and support in times of crisis throughout a six year tenure at the HHC.

## Results

Far from thinking of each other as mere "sex partners," "running buddies" or "drug associates," the 10 couples participating in this study thought of themselves as (common law) spouses, lovers or intimate partners in committed relationships. Most wanted and expected their relationships to endure. Those that didn't were involved in highly conflictive relationships that either dissolved or radically changed during the year-long study or shortly thereafter. Despite often desperate daily struggles, all still aspired to the same social norms that most non-drug users aspire to in their relationships: love, fidelity, material and emotional support, and the ability to maintain a home. These norms often reflected cultural constructions prescribing gender roles and other social practices that were transformed, sometimes dramatically, in the everyday lives of these couples. As a result, these seemingly normative ideals were not always defined in normative ways. Nor were the couples in this study always able to achieve these ideals.

### Caring for each other

One way in which couples attempted to realize conventional gender roles was by taking care of each other. This was true for one couple who limited their drug use without the aid of methadone maintenance (MM), as well as two couples who utilized MM intermittently. These couples clearly derived a sense of satisfaction and security from their relationships. Julio and Sandra, for example, state with no ambiguity the feelings they have for each other, and the ways in which they feel supported in their relationship. Sandra described her feelings for Julio:

S: I'm in love with him. I'm in love with everything he do for me. He understands me, you know ...he's always there for me. We're there for each other during our ups and downs.

Julio described his affection and appreciation for Sandra similarly:

J: I love her. She helps me and I help her. We support each other. Even though there are problems, a marriage that doesn't have problems isn't a marriage.

Sandra also credited her relationship with Julio for saving her from prostitution and the streets.

S: At least I'm not using that much. At least I'm not in the streets. I'm just one man's woman. I know a lot of people used to talk, "There she goes...." That used to bother my son. I'm glad I'm out of the street. I would've end up dead, raped, in jail, or with a bad disease.

Julio, she insisted, also helped her think differently about intimate relationships.

S: Julio taught me that love is not just beating me. My ex used to beat me, then he used to hug me and kiss me and tell me he was sorry. I thought that was O.K. but never again am I gonna let no man lay a hand on me or treat me like shit, because I'm not shit, I'm worth a lot. I was afraid to leave the relationship with my youngest son's father. I thought I couldn't go on without him. He made me feel like if I break up with him I'm not gonna be able to live. That's bullshit. I'll live and I'll keep living.

Sandra's ability to recognize her own worth was clearly made easier by the way in which Julio helped her deal with regret and shame:

J: I have helped her a lot. I tell her, be positive, don't fear anyone, walk with your head held high. What you were, leave it behind you. Let it go because no one is a saint in this world.

Julio also enumerated the ways in which Sandra cared for him by cooking for him when they had food, maintaining their apartment and being attentive to his behavior so she could intervene during episodes of diabetic shock. Sandra has saved Julio's life on three occasions: *J*: *If it weren't for her*, *I wouldn't be here*.

Glenn and Diana, one of the seven couples who rode a roller coaster of moderate to high drug use, also valued and demonstrated care in their relationships. They described how caring among drug-users under these circumstances is not a trivial matter. In a jointly constructed narrative common to long-term couples (they had been together for 6 years), they discussed the bond they share, made all the more "tight" by what they have suffered through as heroin and cocaine addicts with AIDS.

D: Me and Glenn just came tight and we never thought about breaking up. We never even broke up since we've been together.

G: We never even looked...

D: ... in another direction or even thought of breaking up. Never even came cross our minds.

J: What is it that makes you click?

G: It's the caring.

D: We understand one another. And we know the pain that each one of us has felt.

G: We understand, and that just draws me closer to her. She understands what I be going through. It's not like, I'm sick and she ain't or she sick and I ain't. We both going through it.

D: Yeah, not just being sick. All of the reality, everything. We understand everything about each other.

Caring in the context of "all the reality" for the couples in this study meant understanding: the lure of drugs, the pain of addiction and withdrawal, the threat of arrest, fear of separation due to incarceration, and hustling for drugs (panhandling, picking up cans, robbing, muggings, drug selling, or exchanging sex for money or drugs). It also meant coping with persistent poverty, intermittent homelessness, chronic illness (diabetes, epilepsy, hepatitis B and C, HIV/AIDS, depression and anxiety, PTSD), and the stigma attached to addiction, AIDS, and prostitution. It meant grieving for family members and friends who died of hepatitis C, AIDS, overdoses, homicide, as well as children lost to the state or other family members.

### Collusion in managing addictions

Managing addiction, at its most basic level, is about avoiding withdrawal symptoms from heroin and other illicit drugs. Withdrawal from heroin is an extremely painful process that drug users go to great lengths to avoid. To stave off withdrawal symptoms, drug users engage in activities to accumulate the money to buy drugs, obtain the drugs and then share these drugs with their intimate partners. For the couples in this study, these activities necessitated a practical collaboration or collusion to ensure a constant and adequate supply of heroin and other drugs for both partners.

Without exception, every couple pooled resources to obtain drugs, and, when possible, to pay for basic necessities, like rent and food. Strategies to affect this purpose ran the gamut from legal hustles to illegal ones. Julio and Sandra, for instance, spent all of their time collecting recyclable cans to manage their addictions. They used money from Julio's workman's compensation and food stamps to pay for their other basic needs. Other couples also received money from federal assistance programs (e.g., V.A. benefits) or were able to hold temporary jobs. Couples rented rooms or apartments when money was available, but most were evicted when this money was used to support their addictions. Most couples sold drugs or led other drug users to other low-level dealers. Although a gendered division of labor around selling and especially "copping" (buying drugs) was generally maintained, with men selling and/or procuring the drugs for themselves and their partners, and women staying out of sight, this was not always the case. Some couples managed their addictions by forming drug-selling partnerships. These gendered dynamics were often quite fluid. Andrés described how he and Patricia formed a drug-selling partnership after a long period when he was the only one who was selling.

J: Is she selling drugs too?

A: Yeah, she helps me.

J: Ok, so you really are working like partners.

A: Yeah, sometimes she gets it better than what I could get it. She'll get it and she'll give it to me.

J: So is there an advantage to working together?

A: Yeah, cause like that she watches over me. You know I watch over her.

J: So it's mostly like a safety thing.

A: Yeah, you know, 'cause she'll hold the bags. She'll be like in a safe place. I tell people that I got it, but I don't have it on me. I'll alert the people I don't have it on me. If the cop comes and search me, he is not going to find nothing on me.

J: So let's say I want a bag. Are you going to go to her and bring me the bag?

A: Yeah, she'll either come to me, or I'll go to her. Whatever is easier, 'cause I don't want her to get hot, she don't want me to get hot. Or I'll just send the person to her. "You see that person over there, you go over there and she gonna give it to you." And I would look out for the cop, make sure the cop is not coming.

The ultimate pay-off for selling and buying drugs is using them. Meanings such as "share and share alike," "what's mine is yours," and "sharing 50/50" were commonly expressed. Sharing drugs was a cornerstone in these relationships and most couples shared drugs only with each other. This was possible when drug use was at low to moderate levels. Couples with high levels of drug use were not always able to pass up an opportunity to use drugs with others if they were offered. Yet, all 10 couples expressed the ideal of sharing only with each other, even if this ideal could not always be honored. In addition, conflict arose when drugs were not shared equitably among couples, or when one partner attempted to limit his or her drug use while the other's use was at a moderate to high level.

### A dynamic of care and collusion

Care and collusion are part of a dynamic which bonds drug-using couples together in what is often a mutually reinforcing cycle of addiction. We recognize that collusion has a negative connotation suggesting judgment, much like the terms it replaces (i.e., co-dependency, enabling). It is not our aim to denigrate the often desperate activities of drug users, but to highlight the ways in which caring for a partner in the context of an intimate relationship takes place within a particular interpersonal dynamic. Care, which denotes the positive feelings individuals have for each other, as well as the positive ways in which couples interact, and collusion, which denotes the ways in which the bulk of their activities revolve around the need to maintain their addictions, comprise this dynamic. This dynamic usually (but not always) keeps couples spiralling in a cycle of addiction that they themselves describe and experience as an unrelenting form of social suffering.

Couples recognize this dynamic. They acknowledge the synergistic effect of their joint drug use. Juan confessed the nagging guilt he felt about Daisy's heroin addiction. He had recently overheard someone tell Daisy that she'd be better off without him. This harsh judgment unsettled him. Juan and Daisy have been together 11 years and have five children.

If I am the problem then I oughta get help, that way I don't have to low her in, because everybody around us seems to be thinking that I'm the problem because she's into dope. I'm starting to believe that because I have a lot to do with the control of our relationship. She loves me and she's willing to do anything I would do. I go out there and I'm selling the drugs. I'm coming back home with money and the drugs is on me. I had to sell to just use the needle on me. She's gonna see and she wants it. She knows she's sick. She can't do nothing but do this needle just to get normal. And I'm the one who provides the needles for her.

Dealing enabled Juan to supply drugs for both of them, to help pay the rent (when they were housed), and to maintain the respect and dignity that he did not feel he could get from a low-paid, low-status job. He believed selling drugs was his only realistic option.

*But people around her are starting to say that she's better off alone than with me. So that's why I'm strongly looking over *[treatment options]. *If she can hold on, and stay in a place as long as I can go to detox or whatever, I wanna do it. But she's not gonna want to stay alone 'cause I already tried. I mean, lots of times we argued and I ran out the house and didn't wanna come back and she starts crying. So I don't leave.*

Janie: What are you arguing about now?

*Juan: That I be out late. She doesn't want me to get caught with the cops. She says that I'm gonna leave her *[go to jail] *again. She says, ain't no need for me to do it.*

Janie: To be selling?

*Juan: Mmhmm. Which is true because.... I wanna go back to school but right now I am not gonna get caught in no McDonalds and I'm not gonna get caught in no Walgreens or nothing like that. Jobs that I want is not out there or not available for me yet. I need education maybe, but I wanna be a security guard. I can't do that until about four or five years. Or at least I wanna be going to some kind of job with a suit and tie, or a tie and some baggy pants. I don't wanna be going to no Mcdonalds, it's just not me. If it's not out there what I want then, I'm just gonna have to take the risk in doing what I do*.

Juan and Daisy were caught in a perpetual cycle of care and collusion until a major event, like his inevitable arrest, would change this dynamic. Juan accurately recognized that the interpersonal dynamics which propelled their addictions were shaped by larger structural forces – a lucrative drug market in Hartford, the lack of jobs which pay a sustainable wage, the stigma of being an ex-felon, not to mention his (and her) status and identity as a *tecato *(Spanish) or heroin addict. Nevertheless, Juan also recognized that his ability to alter the impact of these larger structural forces was constrained.

Juan's daily life, like that of drug users in general, was continually consumed by the search for "the cure." For this reason, it is difficult to invoke care without also invoking collusion. Couples care for each other by helping each other avoid the symptoms of withdrawal. A loved one is "sick," the partner provides the "cure." Glenn described this practice.

G: I care so, I care so much, you know. And our relationship- as far as drugs go- I'd go to any extreme to help her, to keep her from getting sick. She'd do the same for me; at least I've got that feeling.

Diana concurred. When asked what a typical day is like for her, she replied:

D: Waking up needing a bag of dope. Go to the church for breakfast, that's after I have it. If I don't have it, it ain't a typical day 'cause there ain't no getting up until I get it.

J: So if you can't get up what happens?

D: Stay there, praying.

J: Does Glenn go get it?

D: Yeah. ... He just don't wanna see me like that. He don't wanna see me sick. If you let a person there suffer like that, I don't think... I don't know. I don't know.

### Gendered dynamics

Glenn would go to great lengths to acquire drugs to alleviate Daisy's symptoms of withdrawal. While he stated that "she'd do the same for [him]," this was less likely given the fact that Glenn and Diana (like most of the couples in this study) practiced a gendered division of labor in which men were expected to do the "copping" (acquiring drugs, usually from street dealers). Alternatively, both men and women "copped" together. In the latter case, women were either as actively involved as the men in the kind of partnership noted above, or they went along to make sure their men were safe. In any event, women rarely "copped" alone.

This practice of gendered copping is also perceived by couples as an act of caring. Men cop to assume the risks of arrest and assault. In this sense, gendered copping is considered protective of women. Men take on traditional gender roles of providing and protecting. Several men in this study remarked on this practice. Julio declared, for example: "*I take risks for her because I am her man*". In so doing, he felt a heightened sense of self-respect. In turn, Sandra felt cared for and protected.

In a striking tale, Leonardo and Candy recounted how Leonardo copped for her even though he was "clean." After Candy's cocaine relapse, the state-funded methadone clinic began to lower her methadone dose so she could be dropped from the program. She would not be permitted to re-enroll for several months. During this period, Leonardo explained his behavior: "*I had to quit my job because I didn't want her to be out there, a woman, being out there*". Incredulous, the interviewer asked:

J: *So how did it help, you quitting your job?*

L: I hadda quit it, because I didn't want her being out there like that, hustling the street, I hadda be with her cause I'm her man.

C: Cause I was... coming off methadone is like... it's murder. It really is!

L: Every day I hustled for her. It was very hard, we lost our apartment, we lost everything. We slept in the woods... in the middle of winter.

C: I would wake up sick in the morning and freezing.

L: Rain. It was hard.

C: It was a nightmare.

In this case, it could be argued that Leonardo welcomed Candy's relapse because it provided him a convenient excuse to use again. Regardless, both Leonardo and Candy understood this common practice of men assuming the risks of arrest and assault on the streets as a caring act – the way a man takes care of a woman.

Couples who were best able to maintain stability in their relationships tended to work together to acquire the money for drugs either through drug selling or less risky ventures. When men assumed most of the risks of hustling, as well as copping, conflict often arose in the relationship. Tension mounted between Andrés and Patricia during a period when he was the sole provider of money and drugs, and violence often ensued. He worked for a landscaper 5 or 6 hours a day, did all the copping, and injected Patricia. He supplied her drugs, but not money, and she often had to wait, dope sick, all day until he finished work and acquired her fix. He became more and more exasperated with the double burden of acquiring drugs for himself and for Patricia at a time when he was attempting to limit his own drug use. As a result, he began to blame Patricia for keeping them both in a cycle of addiction. Patricia objected to being blamed.

A: In another week, that's it for me. I'm just gonna stop. Even if she don't stop, I'm gonna stop and ...

*P: I'm gonna stop, I told you *[turning to the interviewer], *we argue.*

A: When I tell her, 'Don't you wanna stop?' she seems like she's not understanding me sometimes.

P: And I feel the same way about him, so.

A: I wanna really stop! You know, I wanna have a car...

P: In other words he's telling me that I don't wanna stop. And I'm telling him the same thing, so we argue.

A: She thinks I'm blaming her. But I tell her, I'm not blaming you because I do it because I want to. But it would be a lot easier for me if I don't have to buy it for you.

J: Is that how it works? Are you doing most of the copping?

A: I do all of it.

P: He do all. I got no kind of income. I depend on him.

A: I work. Look, I work, and I'm so tired of it! And I wanted her to see it. I wanted her to just see the way we are. Just because we in my mother's house, it seems like we're doing good, but we're not.

Patricia did ease Andrés's burden by leaving him. She went to live with one of her sons and made many attempts to enroll in a methadone maintenance program. Initially she was thwarted but, after many efforts over several months, she was finally able to enroll in one of Hartford's two programs. At first, Patricia had no interest in returning to Andrés. She was bitter about the violence she had suffered at his hands, as well as the memory of what she experienced as unnecessary and prolonged dope sickness. Eventually, she did return to him, but on her own terms. For a while, she still refused to live with him, but allowed him to accompany her on hustling and copping escapades. In the end, she proved to be the better provider because of her greater skill at selling drugs.

The dramatic role reversal described above was made all the more poignant when Patricia also began to supply Andrés with drugs and half of her clinic-supplied methadone, something he had done for her in the past. This was not a simple undertaking because methadone is provided in liquid form. Clinic staff watch patients swallow the drug before they are allowed to leave. This type of collusion undoubtedly served both of their purposes. Perhaps Patricia found it hard to stop using heroin completely or didn't want to do so. By sharing methadone with Andrés they could both stave off the pain of withdrawal with a lesser quantity of drugs and still experience a heroin high.

## Discussion

While several of the couples in this study were able to limit their drug use and invest more time and energy into the quality of their relationships, they still lived under the ever-present threat of increased drug use or, if they were able to quit using drugs, of relapse. Others, with more pronounced drug habits, especially polydrug users who used alcohol and crack as well as heroin, colluded in ways most health professionals would consider "dysfunctional."

Despite the constant focus on securing and using drugs in the relationships described above, there are clearly aspects or dynamics in the majority of these relationships that were functional and/or adhere to the same kinds of social norms that many non-drug-using couples aspire to in their relationships. Caring for a partner is one such norm. While care is a common-sense concept when it refers to feelings of affection, romance or benevolent actions that assist a loved one, the claim that drug-using couples care for each other by colluding to acquire and use drugs, is not. This broader definition of care runs counter to dominant theoretical models based on co-dependency or enabling constructs as well as popular notions of responsible and healthy behavior. The idea that one should acquire illicit or even legal psychotropic drugs for a loved one who is suffering the pain and misery of dope sickness or to acquire drugs for a partner to prevent this recurrent outcome does strike drug-users as common-sense, however, and coalesces with their understandings of caring in an intimate relationship.

All twenty men and women in our sample emphasized the importance of sharing drugs in what were often gendered patterns of procurement and use (but mostly procurement). Patterns of use differed in this study from those described in McRae and Aalto's [25] examination of patterns of injecting and needle sharing among women IDUs in Scotland. In their study, women deferred to men's injecting "expertise" which served to place them in a subordinate position in terms of gender hierarchies for HIV risk; namely in their study, men tended to inject themselves first. In our study, by contrast, only Patricia assumed this kind of partner-imposed subordinate role. Ultimately, however, she opted out of this arrangement and forged a new one that offered greater freedom. It could be argued, however, that this new arrangement offered heightened risks. Patricia's options for treatment and counseling, as well as broader life-sustaining supports, such as financial incentives to stay clean and housing, were limited or non-existent right from the start.

Our study also differs from McRae and Aalto [25] insomuch as the men in this study appeared to be as emotionally invested in and committed to their relationships as the women, even when couples were embroiled in conflictive relationships. Most men, like the women, clearly believed that they derived benefits from these couplings; they felt loved and cared for, they felt understood, and they valued the companionship of their partners. When women also helped in the procurement of money and drugs, the males experienced a diminished "burden of care." Our findings indicate that a narrow focus on the way in which power is distributed in relationships, while important, does not provide us with an adequate understanding of intimate relationships among drug users – there are other ties that bind. When couples were able to maintain sobriety, enrolled in methadone programs, or otherwise limited their drug use, like Sandra and Julio succeeded in doing, they were able to focus more on the quality of their relationships. This, in turn, also provided needed motivation for continuing in their efforts to get clean or reduce drug consumption and attend to other health needs, such as the need for adhering to HIV medication regimes. In these cases, couples supported each other by creating and maintaining strategies that greatly benefited both partners. Their dependence on heroin and cocaine was reduced and their overall quality of life improved. The potential for this turn of events among drug users is obscured by the presumption of dysfunctionality and merits attention.

## Conclusion

Existing studies tend to view sexual relationships between drug-using couples as dysfunctional, unstable, utilitarian, and often violent. While the findings from this study do not wholly contradict these perceptions (i.e., some relationships, or some relationships at certain times, were dysfunctional, unstable, utilitarian and/or violent), our findings – while admittedly based on a small sample – suggest that a more complex and nuanced understanding of drug-using couples is needed. A more realistic portrayal needs to include the following: 1) a recognition of the heterogeneity which exists in the types and quality of intimate relationships among drug users; 2) as well as the ways in which these relationships are valued for more than the material benefits they provide (pooled resources, including drugs) by the high proportion of drug users who participate in them.

In addition, our long-term experience with many of these couples enabled us to recognize that relationships were far from static. Interpersonal dynamics changed in response to altered circumstances (i.e. frequent incarcerations) and individual agency (i.e. leaving an abusive relationship). Gendered dynamics revealed themselves to be especially fluid. What hasn't changed, except for the worse, is the need for expanded social and treatment services to assist impoverished drug users with poor education and meager job experience or skills, to reduce the harms associated with decades-long dependence on heroin, cocaine and crack, as well as other health risks (see Singer [51] and Chien et al [52] for a discussion about why it is easier to get drugs than drug treatment in the U.S.).

Left primarily to their own survival strategies, drug users manage their addictions and other health risks the best way they can: by depending on each other. Those who were able to reduce risk often utilized a strategy of social isolation, limiting their contacts to kin or each other. In these cases, for partners who had been able to avoid infection prior to establishing relationships with their partners, their relationships provided protective benefits from HIV. (However, because none of the couples used condoms, this strategy could very well have enabled HIV transmission in serodiscordant couples. Indeed, two of the HIV+ women in this study believed they were infected by their current partners.) Gendered dynamics in otherwise supportive relationships also provided protection from arrest and street violence for women. These dynamics cannot be adequately understood without considering the meaning of risk and relationship for drug-using couples.

Researchers have begun to recognize the influence that intimate partners have in perpetuating cycles of drug use. Our study offers one partial explanation by demonstrating the ways in which gendered dynamics, such as the care and collusion dynamic, tend to maintain addictions and perpetuate cycles of increasing drug use. We also have shown, however, that some couples were able to overcome this tendency, despite great odds, and reduce drug use or abstain from heroin and other substances in supportive relationships. It is important to emphasize, however, how interpersonal dynamics, such as the care and collusion dynamic, are shaped by and interact with larger structural forces, including structural limitations in the treatment system, which constrain access to essential health and risk reduction services.

Our findings have direct implications for drug treatment programming. The lower rung of the severely under-funded two-tiered drug treatment system in the U.S. (a private system for those who can afford it, a narrow public system for those who cannot) does not provide even basic services to the intimate partners or family members of drug users. Indeed, in drug treatment, such relationships are often seen as a threat to recovery, as they have the potential to pull patients out of treatment and back onto the streets with a resumption of drug use. Notably, in other branches of therapy, romantic relationships are not only recognized but routinely are built into the therapeutic process both conceptually and in terms of available services (e.g., marriage counseling, couples therapy, family therapy). By contrast, illicit drug dependence and addiction tends to be seen and treated as an individual problem, as if drug users were not capable of having romantic relationships, and certainly not romantic relationships that are supportive and caring.

In order to assist couples to achieve positive, life-enhancing results, treatment programs need to acknowledge the existence and importance of these relationships in the lives of drug users by advocating treatment program policies and practices which support this understanding. They then need to work with couples, rather than against them, in the provision of a wide array of couples-focused intervention services, including residential and outpatient couples drug treatment and relationship counseling, aftercare support, and couples relapse prevention, as well as supportive services for housing, education, job training and placement services. Understandably, this change has the greatest implications for residential therapy, which tends to be gender segregated. It should be noted that drug treatment systems were, in the past, resistant to recognizing that drug users can be pregnant and have children. In response to community pressure, however, there has been significant progress made in the development of residential drug treatment services for pregnant women that accommodate, as well, their other children.

In addition, the lives of these couples, like the lives of other impoverished drug-users, underscore the necessity of ameliorating the large-scale structural forces that drive drug-selling and drug-use and make decades-long addictions all the harder to overcome. A serious commitment to address the drug problem – one that has not been successfully addressed by emphasizing the arrest and imprisonment of chemically dependent individuals – will require the development of an integrated and comprehensive drug treatment system which includes and seeks to support romantic relationships as a potential resource in recovery.

## Abbreviations

HCV: Hepatitis C

## Competing interests

The author(s) declare that they have no competing interests.

## Authors' contributions

JS recruited and interviewed study participants, conducted ethnographic observations, coded and analyzed transcripts, and wrote the first draft of the manuscript. MS was consulted on the study design and collaborated in the writing of subsequent drafts of the manuscript.
